# Tissue Tolerable Plasma (TTP) induces apoptosis in pancreatic cancer cells *in vitro* and *in vivo*

**DOI:** 10.1186/1471-2407-12-473

**Published:** 2012-10-15

**Authors:** Lars Ivo Partecke, Katja Evert, Jan Haugk, Friderike Doering, Lars Normann, Stephan Diedrich, Frank-Ulrich Weiss, Matthias Evert, Nils Olaf Huebner, Cristin Guenther, Claus Dieter Heidecke, Axel Kramer, René Bussiahn, Klaus-Dieter Weltmann, Onur Pati, Claudia Bender, Wolfram von Bernstorff

**Affiliations:** 1 Department of General, Visceral, Thoracic and Vascular Surgery, University Medicine Greifswald, Ernst-Moritz-Arndt University, Greifswald, Germany; 2 Institute of Pathology, University Medicine Greifswald, Ernst-Moritz-Arndt University, Greifswald, Germany; 3 Department of Gastroenterology and Nephrology, University Medicine Greifswald, Ernst-Moritz-Arndt University, Greifswald, Germany; 4 Institute of Hygiene and Environmental Medicine, University Medicine Greifswald, Ernst-Moritz-Arndt University, Greifswald, Germany; 5 Leibniz Institute of Plasma Science and Technology (INP), Felix Hausdorff-Str. 2, Greifswald, Germany

**Keywords:** Tissue tolerable plasma (TTP), Plasma medicine, Apoptosis, TUM-CAM Assay, Pancreatic cancer

## Abstract

**Background:**

The rate of microscopic incomplete resections of gastrointestinal cancers including pancreatic cancer has not changed considerably over the past years. Future intra-operative applications of tissue tolerable plasmas (TTP) could help to address this problem. Plasma is generated by feeding energy, like electrical discharges, to gases. The development of *non-thermal atmospheric plasmas* displaying spectra of temperature within or just above physiological ranges allows biological or medical applications of plasmas.

**Methods:**

We have investigated the effects of tissue tolerable plasmas (TTP) on the human pancreatic cancer cell line Colo-357 and PaTu8988T and the murine cell line 6606PDA *in vitro* (Annexin-V-FITC/DAPI-Assay and propidium iodide DNA staining assay) as well as in the *in vivo* tumour chorio-allantoic membrane (TUM-CAM) assay using Colo-357.

**Results:**

TTP of 20 seconds (s) induced a mild elevation of an experimental surface temperature of 23.7 degree Celsius up to 26.63+/−0.40 degree Celsius. *In vitro* TTP significantly (p=0.0003) decreased cell viability showing the strongest effects after 20s TTP. Also, TTP effects increased over time levelling off after 72 hours (30.1+/−4.4% of dead cells (untreated control) *versus* 78.0+/−9.6% (20s TTP)). However, analyzing these cells for apoptosis 10s TTP revealed the largest proportion of apoptotic cells (34.8+/−7.2%, p=0.0009 *versus* 12.3+/−6.6%, 20s TTP) suggesting non-apoptotic cell death in the majority of cells after 20s TTP. Using solid Colo-357 tumours in the TUM-CAM model TUNEL-staining showed TTP-induced apoptosis up to a depth of tissue penetration (DETiP) of 48.8+/−12.3μm (20s TTP, p<0.0001). This was mirrored by a significant (p<0.0001) reduction of Ki-67+ proliferating cells (80.9+/−13.2% *versus* 37.7+/−14.6%, p<0.0001) in the top cell layers as well as typical changes on HE specimens. The bottom cell layers were not affected by TTP.

**Conclusions:**

Our data suggest possible future intra-operative applications of TTP to reduce microscopic residual disease in pancreatic cancer resections. Further promising applications include other malignancies (central liver/lung tumours) as well as synergistic effects combining TTP with chemotherapies. Yet, adaptations of plasma sources as well as of the composition of effective components of TTP are required to optimize their synergistic apoptotic actions.

## Background

The development of multimodal therapies for different tumour entities along with highly specialized radical tumour resections have greatly improved the outcomes in many malignancies [1,2]. However, in gastrointestinal cancers in general and in pancreatic cancer in particular the rate of microscopic incomplete resections has not changed considerably over the past few years. Frequently, in pancreatic cancer surgeons are left in this dilemma of in situ remaining tumour cells [3,4]. The future intra-operative application of tissue tolerable plasmas could help to address this problem in a clinical setting. Special applicators of plasmas could help to destroy microscopic residual tumour cells of retroperitoneal tissues as well as on central coeliac and mesenteric vessels. This could greatly increase radical resections improving the prognosis of pancreatic cancer at the same time.

In physical sciences, the term “plasma” refers to the forth state of matter [5]. It is generated by feeding energy, e.g. electrical discharges, to gases [6]. Plasma itself consists of positively and negatively charged particles (i.e. ions and electrons), free radicals, neutral and excited atoms or molecules as well as photons. In all plasmas supported by electric field electrons receive the external energy much faster than the much heavier ions and have the opportunity to heat up to several thousands of degrees before their environment heats up [7].

In non-thermal plasma, cooling of ions and uncharged molecules is more effective than energy transfer from electrons and the gas remains at low temperature. In thermal plasma, on the other hand, energy flux from electrons to heavy particles equilibrates the energy flux from heavy particles to the environment only when temperature of heavy particles becomes almost equal to the electron temperature [6] and [7]. The development of *non-thermal atmospheric plasmas* displaying spectra of temperature within or just above physiological ranges, i.e. tissue tolerable plasmas (TTP), has made the use of plasmas possible for biological or medical applications [8,9]. This includes the utilization in living organisms. First results on the compatibility and application of plasmas have been gained in the fields of dermatology as well as hygiene and microbiology [10-14]. In addition, initial reports on the efficacy of plasmas on tumours have been published [6,15].

In this study, we have investigated the effects of tissue tolerable plasmas (TTP) on the human pancreatic cancer cell line Colo-357 *in vitro* as well as in the *in vivo* tumour chorio-allantoic membrane assay (TUM-CAM assay).

## Methods

### Cell line and culture

The human pancreatic adenocarcinoma cell line Colo-357 (established by Morgan et al. [16]) was maintained in RPMI-1640 medium supplemented with 10% fetal calf serum, 100 U/ml of penicillin and 100 μg/ml of streptomycin (referred to as “complete medium”). In addition, the murine pancreatic cancer cell line 6606PDA [17] and the human pancreatic cancer cell line PaTu8988T [18] were tested *in vitro* to exclude cell specific effects of TTP. 6606PDA cells were cultured in complete medium, PaTu8988T cells were cultured in DMEM high glucose, supplemented with 10% fetal calf serum, 100 U/ml of penicillin and 100 μg/ml of streptomycin. Tissue culture reagents were obtained from Gibco (Invitrogen, Carlsbad, California, USA). Cell cultures were kept pathogen-free in a humidified incubator at 37°C with 5% CO_2_. Cell cultures were regularly tested for *Mycoplasma* species. They were consistently negative for mycoplasma contamination.

### Tissue tolerable plasma (TTP)

**Figure 1:**
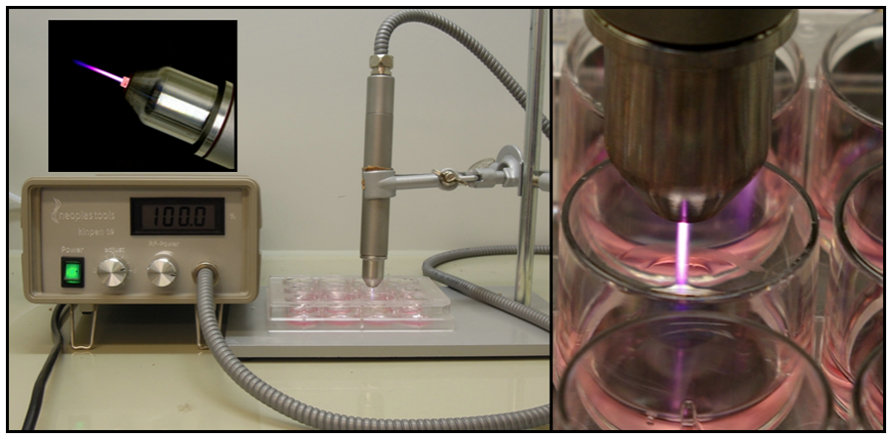
**Plasma jet kINPen09 (Neoplas GmbH, Greifswald, Germany) - application of TTP *in vitro.***

The plasma was generated with the atmospheric pressure plasma jet kINPen09 (Neoplas GmbH, Greifswald, Germany, CE certification No. 609.003.1, Figure 1) as previously described [9] argon being the carrier gas. The plasma was used in continuous mode with following settings: gas flow 4 standard litres per minute (slm); supply voltage Usupl. = 65 V DC (system power: 8W at 220V, 50/60Hz); frequency f = 1.1 MHz. In continuous mode the plasma temperature was 45°C and the length of the effluent plasma was 11 mm measured from the nozzle.

### Measurement of temperature induction of TTP

The surface temperature of cell culture dishes was measured after 5, 10 and 20 seconds of TTP treatment using a Voltcraft K102 digital thermometer (Conrad Electronic SE, Hirschau, Germany) employing a paddle surface probe with a miniature plug (B+B Thermo-Technik, Donaueschingen, Germany). Measurements were repeated 10 times in each case.

### Plasma treatment and detection of cell viability and apoptosis *in vitro*

Colo-357 cells were grown to subconfluency, harvested using a 0.05% trypsin solution, washed twice in PBS, resuspended in fresh medium and counted. Cell culture inserts (5mm plastic templates, self-made) were used in 12 well plates (BD Bioscience, Heidelberg, Germany) to grow tumour cells for plasma treatment. 1x10^6^ Colo-357 cells were added into cell culture inserts and grown in complete medium for 24 hours. Then, the medium and the plastic templates were removed leaving only a thin liquid film of medium on the tumour cells. Plasma treatment was carried out using the above described plasma jet kINPen09. The plasma jet was applied for different time periods, i.e. 5 seconds, 10 seconds, and 20 seconds. The plume was guided randomly in a meandering course across the tumour cells. To ensure optimal plasma effects on the tumour cells the distance from the nozzle to the tumour cells did not exceed 10 mm. One control group was treated with argon gas only for 20 seconds; one control group was left untreated. After treatment cultures received fresh medium. Tumour cells were analyzed 1, 24, 48, and 72 hours following plasma treatment. Analyses were performed using a propidium iodide DNA staining assay, using the TREG-detection kit (Miltenyi Biotec GmbH, Bergisch Gladbach, Germany) for intracellular staining of FOXP3 (forkhead box P3). After a 15 minute incubation step with the permeabilization buffer cells were centrifuged and resuspended in 100 μl of this buffer adding 30 μl of RNase A (1mg/ml, R4875, Sigma-Aldrich, Hamburg, Germany) and 1 ml of binding buffer consisting of HBSS (Sigma-Aldrich, Hamburg, Germany), 10% FCS (PAN™ Biotech GmbH, Aidenbach, Germany) and 0.01 M HEPES (Biochrom, Berlin, Germany). This was followed by 15 minutes of incubation at 37°C. Then, 200 μl of propidium iodide (25μg/ml) were added, cells incubated for 30 minutes at room temperature and washed in binding buffer. Cells were resuspended in 100 μl of binding buffer followed by flow cytometric analyses. In a second test an Annexin-V-FITC/DAPI-Assay was employed. Briefly, tumour cells were released from the plastic surface with 300μl EDTA (10mM), washed in medium followed by washing in binding buffer. Then, cells were resuspended in 100μl of binding buffer adding 1 μl of FITC-Annexin-V (BD Pharmingen, Heidelberg, Germany) followed by 30 minutes of incubation at 4°C. Afterwards, cells were washed in binding buffer and resuspended in 150 μl of binding buffer adding 1 μl of DAPI (stock was made of 100μg/μl DAPI (Invitrogen**,** Life Technologies, Darmstadt, Germany) diluted with PBS to 1μg/μl). Cells were then analyzed by flow cytometry. Both protocols employed the flow cytometer BD FACS Canto II (BD Biosciences San Jose, USA) analyzing the results with the BD FACS Diva software and FlowJo7.6.4. To exclude cell specific effects of TTP on Colo-357 the cell lines 6606PDA and PaTu8988T were also treated with TTP. After 10 seconds of TTP treatment cells were analysed after 1, 6 and 24 hours using the Annexin-V-FITC/DAPI-staining assay.

### TUM-CAM (Tumour-Chorio-Allantoic Membrane) model

Vakzine Lohmann specific pathogen free eggs (VALO SPF) were obtained from VALO Biomedia GmbH (Osterholz-Scharmbeck, Germany). After delivery the eggs were incubated for 6 days at 37 ± 1°C and 65 ± 7% relative humidity in a small motored breeder (KMB F/2, Ehret GmbH, Emmendingen, Germany). On day 6 a small hole (≤1 mm) was punched into the pointed end of the egg. Then, the hole was closed again with a self adhesive tape. The eggs were placed with the blunt end, containing the air cell, facing downwards into the breeder and incubated for one additional day without further rotation. On day 7 the egg was carefully opened at the pointed end and the surface of the CAM was slightly roughened using a 5x5 mm filter paper (TISSUE-TEK II, Vogel, Giessen, Germany) saturated with diethyl ether. Onto this area a silicone ring was placed, the ring having a 5 mm inner and a 6 mm outer diameter. Colo-357 cells that had been prepared as described above, were resuspended at 2x10^6^ cells in 15 μl of a mixture containing 5 μl RPMI-1640 (without additives) and 10 μl BD Matrigel™ Basement Membrane Matrix (BD Biosciences, Bedford, USA). Thereafter, this 15 μl tumour cell suspension was carefully filled into the silicone ring. The defect in the eggshell was closed with the transparent medical dressing Tegaderm™ (3M Healthcare, Neuss, Germany) to avoid harmful evaporation of the culture. Eggs were then cultured for an additional 5 days in the breeder. Unfertilized eggs were excluded from the study.

### Plasma treatment in the TUM-CAM model

**Figure 2:**
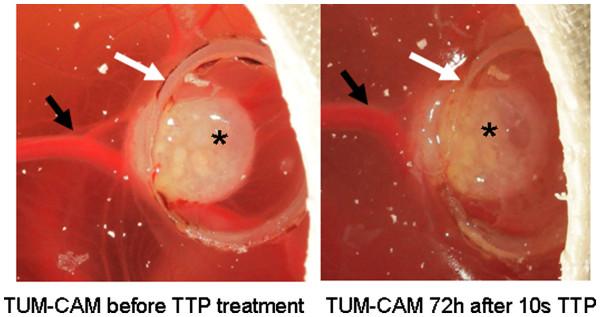
TUM-CAM (Colo-357) before and 48h after 10s TTP treatment (asterisk: tumour; black arrow: supplying blood vessel; white arrow: silicone ring).

On day 12 the grown tumours on the CAM were treated with plasma employing the kINPen09 as described above using the same time schedule and the same technique of application (Figure 2). Two control groups (untreated control and argon gas only) were treated as delineated above. Following plasma treatment the eggs were cultured for further 48 hours, as described above.

### Histology and immunohistochemistry

Tumours were harvested from the CAM using a standard pointed scalpel, fixed with neutral buffered formalin over night and embedded in paraffin. 2 μm sections were cut vertically and mounted on glass slides (SuperFrost®Plus, Menzel GmbH & Co. KG, Braunschweig, Germany). Thereafter, sections were deparaffinized with xylene and 100% ethanol and stained with HE (haematoxylin-eosin) and PAS (Periodic acid-Schiff reaction) according to standard protocols. Fertilized eggs were sacrificed by freezing them at −20°C.

Cell proliferation (Ki-67) was analysed by immunohistochemistry using an automated slide-staining system (Ventana BenchMark XT, Roche, Mannheim, Germany). Antigen retrieval was achieved by heat mediated buffer (pH 9.0) treatment. After rinsing in phosphate-buffered saline (PBS) endogenous peroxide activity was blocked by incubation in 3% hydrogen peroxide. After washing, non-specific binding was blocked with Aurion-BSA (Aurion, Wageningen, Netherlands). Sections were then incubated with the primary antibody (anti-Ki-67, monoclonal Mouse-Anti-Human Ki-67 Antigen Klone MIB1, 1:50 dilution, DAKO, Hamburg, Germany) for 32 minutes at 37°C. The primary antibody complex was visualized using a multimer-technology based detection system (ultraView Universal DAB, Roche, Mannheim, Germany) according to the manufacturer’s instructions. Negative controls contained all reagents except the primary antibody.

The slides were examined using a Keyence BZ-9000 microscope (Keyence, Frankfurt, Germany). For determination of the proliferation index Ki-67 positive cells were counted using the software *dynamic cell count* (BZ-II Analyzer, Keyence, Frankfurt, Germany). Negative cells were manually counted.

### Detection of apoptosis in vivo (TUNEL-Assay)

The number and distribution of apoptotic cells within the explanted micro tumours was analyzed using the FragEL™DNA Fragmentation Detection Kit (CALBIOCHEM, Merck, Darmstadt, Germany) according to the manufacturer’s protocol.

### Statistical methods

Statistical analysis was performed using GraphPad Prism (Version 5.01) for Windows software (GraphPad Software, San Diego, CA, USA). Results were analyzed using the Kruskal-Wallis test, Dunn's Multiple Comparison and the Mann–Whitney test. A p-value below 0.05 was considered to be statistically significant. If not stated otherwise the standard deviation is given.

## Results

### TTP induced experimental mild hyperthermia

In culture dishes equipped with a digital thermometer we found a mild elevation of the original surface temperature (i.e. 23.7°C) of 2.02°C +/0.56°C after 5 seconds of TTP treatment, of 2.57°C +/−0.23°C after 10 seconds of TTP treatment, and 2.93°C +/−0.40°C after 20 seconds of TTP treatment. Multivariate analysis indicates a significant increase of temperature between 5 and 20 seconds of application (p=0.0019). However, testing within the groups using the non-parametric Mann–Whitney test we found significant differences between all groups (p=0.0276 for 5 *versus* 10 seconds, p=0.0243 for 10 *versus* 20 seconds) pointing to possible significant differences if analyzing large sample groups.

### Plasma induced cell death in pancreatic cancer cells *in vitro* as shown by Annexin-V-FITC/DAPI-staining

**Figure 3:**
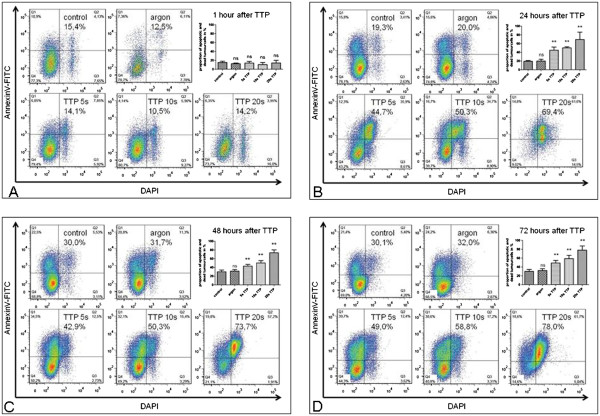
**Plasma induced cell death in pancreatic cancer cells *in vitro *as shown by Annexin-V-FITC/DAPI-staining A: after 1h; B: after 24 h; C: after 48h; D: 72 h after TTP treatment.**

Analyzing *in vitro* grown pancreatic cancer cells 1h after plasma treatment there was no relevant increase of cell death using Annexin-V-FITC/DAPI compared to controls (Figure 3A, Table 1). However, a plasma effect on cell death was detectable 24h after treatment and further increased up to 72h after treatment levelling off thereafter (Figure 3B-D, Table 1). 24–72 hours after Plasma treatment we found a significant increase (1.4- to 3.6-fold) in dead cells compared to untreated or argon gas treated controls. In addition, we identified a correlation of increased death rates with the duration of the TTP treatment. In summary, longer TTP application affects cell viability, but the effect becomes visible only after 24-72h (Table 1). In addition, the murine pancreatic cancer cell line 6606PDA as well as the human pancreatic cancer cell line PaTu8988T were analysed in similar experiments (Additional file 1: Table S1). 6606PDA displayed a high spontaneous rate of cell death. After TTP treatment there was a significant increase of cell death compared to control cells (p=0.0303). Also, PaTu8988T displayed a significant increase of cell death following TTP treatment (p=0.0043).

**Table 1:** **Impact of TTP on cell viability of Colo-357 *in vitro *using Annexin-V-FITC/DAPI**

**treatment**	**1h**	**24h**	**48h**	**72h**
control	15.2% (+/−2.8%)	19.3% (+/−1.8%)	30.0% (+/−3.9%)	30.1% (+/−4.4%)
argon gas	12.5% (+/−1.6%)	20.0% (+/−3.9%)	31.7% (+/−3.4%)	33.0% (+/−4.0%)
5s plasma	14.1% (+/−3.4%)	44.7% (+/−7.5%)**	42.9% (+/−3.9%)**	49.0% (+/−5.9%)**
10s plasma	10.5% (+/−4.7%)	50.3% (+/−2.4%)**	50.3% (+/−5.4%)**	59.0% (+/−7.0%)**
20s plasma	14.2% (+/−5.8%)	69.4% (+/−16.7%)**	73.7% (+/−6.5%)**	78.0% (+/−9.6%)**

** Indicates significant difference compared to controls (P< 0.01).

### Plasma treatment induced apoptosis in pancreatic cancer cells *in vitro* as demonstrated by propidium iodide cell cycle analyses

We analysed the mode of cell death by cell cycle analyses employing propidium iodide. We saw a significant increase of apoptotic cell numbers after Plasma treatment compared to untreated controls as well as Argon controls (for all results compare Table 2 and Additional file 2: Figure S1). Still, there was also a slight but significant increase of apoptotic cell numbers after argon gas treatment compared to untreated controls (p= 0.0013). Plasma treatment for 5 seconds leads to a significant increase of apoptotic cells which was even higher at 10 seconds of plasma treatment. Using 10 seconds of plasma treatment the effects reached a peak after 72 hours whereas 5 seconds of plasma treatment peaked after 48 hours. Also, 10 seconds of plasma treatment lead to a higher apoptotic rate than 5 seconds of plasma treatment. However, this was not statistically significant for the time point 72 hours after plasma treatment (p= 0.0695). Regarding 20 seconds of plasma treatment we detected a slight though significant increase of apoptosis only after 72 hours compared to the untreated control (p=0.0143). However, the apoptosis rate after 72 hours following 20 seconds of plasma treatment was highly significantly smaller than after 10 seconds of plasma treatment (p=0.0009).

**Table 2:** **Plasma treatment induced apoptosis in pancreatic cancer cells *in vitro *(propidium iodide cell cycle analyses)**

**treatment**	**1h**	**24h**	**48h**	**72h**
control	1,8% (+/−0.4%)	1,2% (+/−0.2%)	2,5% (+/−0.7%)	2,9% (+/−1.3%)
argon gas	0,7% (+/−0.3%)	2,1% (+/−0.5%)	6,9% (+/−1.9%)	7,9%* (+/−1.9%)
5s plasma	0,4% (+/−0.1%)	13,3%* (+/−6.2%)	31,1%* (+/−2.9%)	26,0%* (+/−6.0%)
10s plasma	1,8% (+/−3.0%)	17,9%* (+/−2.6%)	31,2%* (+/−9.7%)	34,8%* (+/−7.2%)
20s plasma	0,9% (+/−0.2%)	2,8% (+/−1.4%)	6,2% (+/−4.2%)	12,3%* (+/−6.6%)

* Indicates significant difference compared to controls (P< 0.05).

** Indicates significant difference compared to controls (P< 0.01).

In summary, plasma treatment significantly induces apoptosis in pancreatic cancer cells *in vitro*, with a treatment duration of 10 seconds showing the strongest effect. Interestingly, 20 seconds of plasma treatment displayed only a small induction of apoptosis in this setting.

### Plasma inhibited proliferation and induced apoptosis in solid pancreatic cancer tumours of the TUM-CAM model

**Figure 4:**
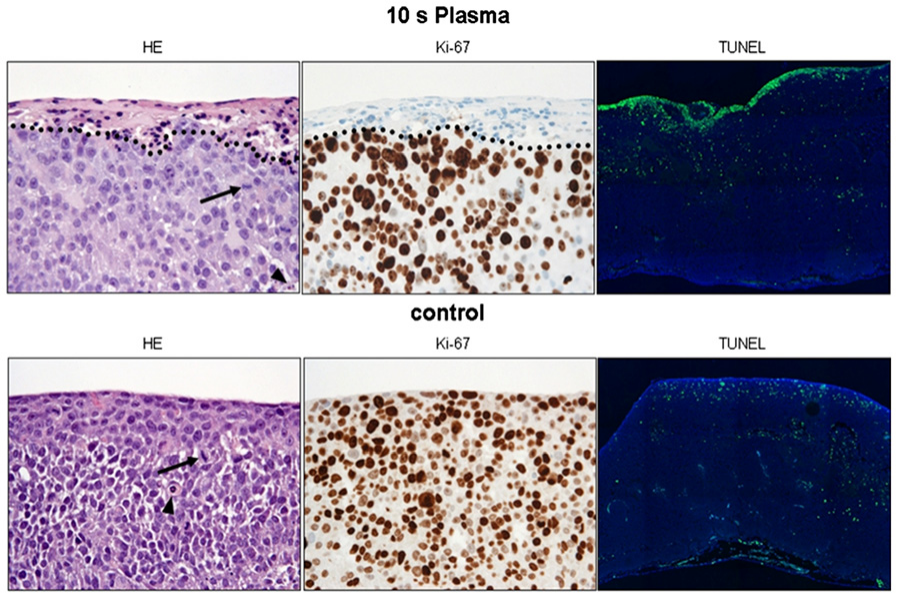
**Histology using HE-, Ki-67-, and TUNEL-staining of the TUM-CAM model after plasma treatment.** Plasma treatment of 10 seconds and 20 seconds (not shown) led to cell death in the upper 3–5 cell layers (above the dotted line), accompanied by a complete absence of viable proliferating cells (Ki-67-staining) and a strong increase in apoptotic activity (TUNEL staining). However, the bottom cell layers were not affected. One mitotic figure (arrow) and a spontaneous apoptotic body (arrowhead) of the unaffected deep layers of a plasma treated tumour as well as an untreated control tumour were marked. The lower edge of HE- and Ki-67-stains measure 250μm (original magnification 400x). Original magnification of TUNEL staining: 200x.

Microscopical analyses of solid tumours using HE stained specimens displayed changes within the upper 3 to 5 cell layers of TTP treated tumours. This was true for all TTP application times (Figure 4). Altered cells showed early microscopic signs of cell death, i.e. cellular shrinking, a condensed bright eosinophilic cytoplasm and pycnotic dark small nuclei as a result of chromatin condensation. No inflammatory reaction was noted. No effects were observed in deeper layers: Similar to the untreated cells in the control, they retained their malignant phenotype and showed mitotic activity as well as some spontaneous apoptotic bodies (HE staining, Figure 4).

**Figure 5:**
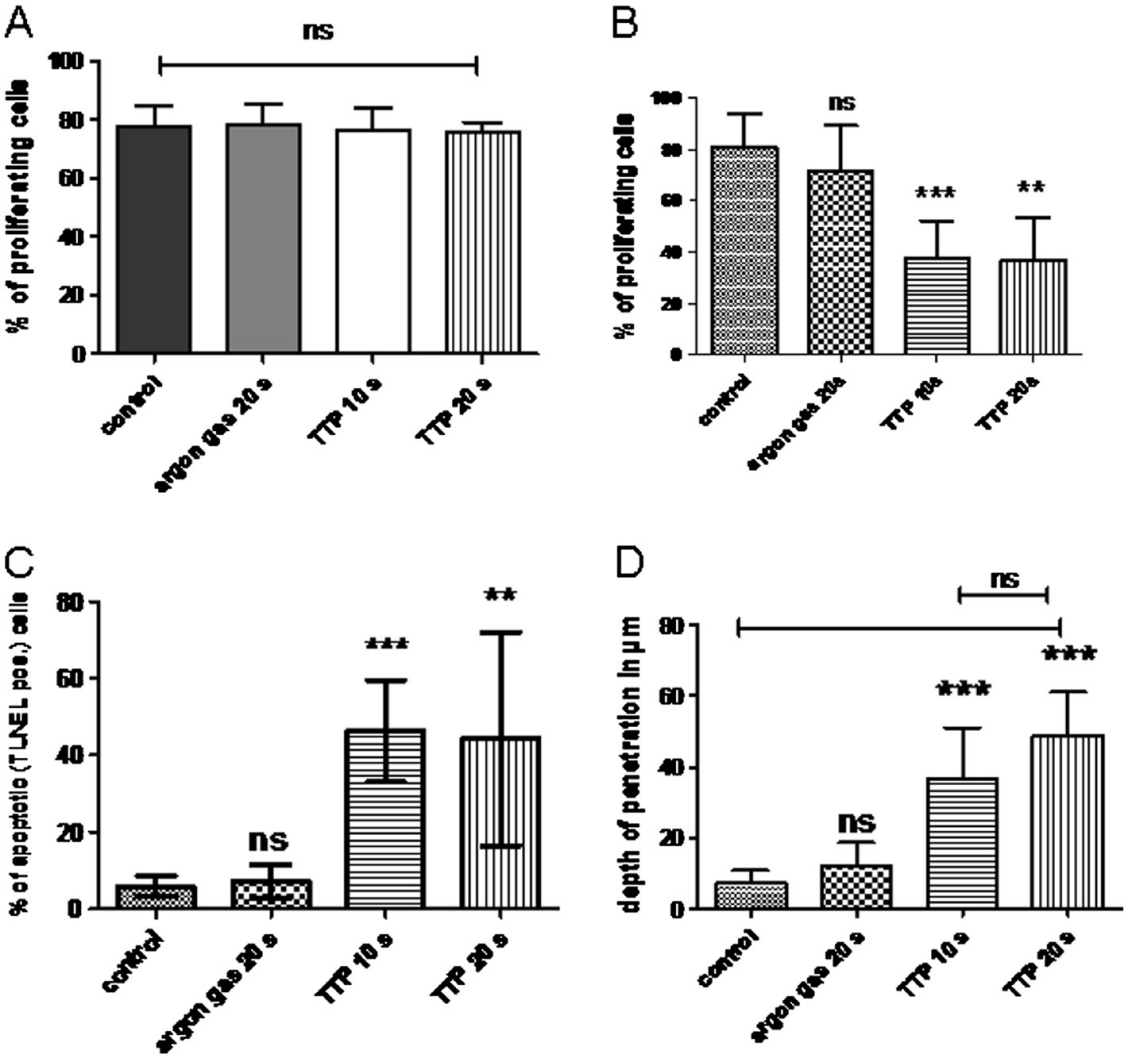
**Impact of TTP in the TUM-CAM model on tumour proliferation and apoptosis A: Using the proliferation marker Ki-67 there were not significant differences in proliferation of total tumour specimens (Figure**5**A, p= 0.9355).B**: Analyzing the upper 100 μm of tumour tissue argon gas had no significant effect on proliferation. 10s TTP highly significantly decreased proliferation (p< 0.0001), with 20s TTP showing no additional effects (10s *versus* 20s p= 0.9359). **C**: TTP induced highly significant apoptosis after 10 seconds (p< 0.0001), and after 20 seconds (p= 0.0006) in the upper 100 μm of the tumour. **D**: Depth of effective tissue penetration (DETiP) of 20s TTP was up to about 60μm (p<0.0001), with 10s TTP not being significantly different (p=0.1428).

Then, we analyzed the number of proliferating cells using the proliferation marker Ki-67. Work-up of total tumour specimens did not bring up any significant differences (Figure 5A, p= 0.9355) between untreated controls (percentage of Ki-67+ cells 77.4 +/− 7.3%), argon gas controls (78.3 +/− 6.7%,), plasma treatment for 10 seconds (76.6 +/− 7.5%), and plasma treatment for 20 seconds (76.0 +/− 3.1%).

However, analyzing the different cell layers of the micro tumours a significant impact of plasma treatment onto tumour cells could be demonstrated. Examination of the tumour layers was then carried out from top to bottom, the top layers having had direct contact with the plasma plume. Corresponding to the histology, the top cell layers displayed almost absent cell proliferation whereas the bottom layers consisted of strongly proliferating cells (Figure 4). More specifically, analyzing the number of proliferating cells in the top 100 μm of the tumour specimens using the proliferation marker Ki-67 did not bring up any significant differences between untreated controls (percentage of Ki-67+ cells 80.9 +/− 13.2%) and argon gas (71.9 +/− 17.6%, p= 0.2321). Yet, we could demonstrate a highly significant effect of TTP on cell proliferation in the top layers of the tumours after 10 seconds TTP (37.7% +/− 14.6%, p< 0.0001) and a significant, but not additional effect after 20 seconds TTP (37.0% +/− 16.4%, p< 0.0001, 10 seconds *versus* 20 seconds p= 0.9359, Figure 5B).

In addition, corresponding to the absent Ki-67 staining, TUNEL analyses demonstrated apoptosis of tumour cells: Untreated control tumours and argon gas only treated controls displayed only single apoptotic cells within the top cell layers whereas plasma treated tumours showed increased zones of apoptotic cell layers at the top (Figure 4). This was true for 10 seconds of plasma treatment as well as for 20 seconds of plasma treatment.

Corresponding to the Ki-67 staining using the TUNEL method we could demonstrate a highly significant induction of apoptosis in the top 100 μm of the tumours after 10 seconds TTP (46.2% +/− 13.3%, p< 0.0001). This could not be further increased after 20 seconds TTP (44.3% +/− 27.8%, p= 0.0006, 10 seconds *versus* 20 seconds p= 0.8617, Figure 5C).

Combining the results of HE staining, immunohistochemistry and TUNEL-staining the depth of effective tissue penetration (DETiP) of the plasma jet could be determined (dotted lines in Figure 4). Following 10 seconds of treatment a DETiP of 36.8 +/− 14.2 μm was found. This was significantly more compared to changes of the untreated and the argon gas only treated controls (7.0 +/− 3.7 μm, and 12.0 +/− 6.7 μm, respectively; p< 0.0001). 20 seconds of treatment showed no significant increase (p= 0.1428) of the DETiP to 48.8 +/− 12.3 μm compared to 10 seconds of treatment (Figure 5D).

In summary, TTP induced a significant reduction of cell proliferation as well as apoptosis in the top cell layers of solid TUM-CAM tumours. This effect was seen up to a DETiP of about 60 μm. The *in vitro* effect of 20 seconds of TTP of non-apoptotic cell death was not present in solid TUM-CAM-tumours showing mainly apoptosis.

## Discussion

In the search of adjuvant treatment options to decrease minimal residual disease after surgery of malignancies in general and pancreatic cancer in particular tissue tolerable plasmas (TTP) could prove a valuable tool. In this study we show that TTP causes cell death in pancreatic cancer cells *in vitro*. Up to 10 seconds of treatment induced apoptosis whereas longer application led to non-apoptotic cell death, all effects levelling off after about 72 hours after treatment. In the TUM-CAM model TTP induced apoptosis only in the top cell layers showing a depth of effective tissue penetration (DETiP) of up to 60 μm. This represents the top five cell layers leaving the lower cell layers intact. TTP has also been named non-thermal [19] or cold plasma [20]. Yet, we experienced small but measurable increases of the temperature following TTP treatment. However, even after 20 seconds of TTP application the temperature increased by a maximum of only 3.5°C. Assuming a body temperature of 37.0°C the expected maximum tissue temperature even after 20 seconds of TTP application would not exceed 41°C. This mild TTP induced elevation of tissue temperature is rather unlikely to exert negative effects on surrounding healthy tissues in tumour patients [21]. Similar *in vivo* results in mice growing subcutaneous melanomas were demonstrated by Keidar et al. in 2011 [21].

The treatment of pancreatic cancer cells *in vitro* using TTP induced non-apoptotic cell death as well as apoptosis. To exclude cell line specific effects of the pancreatic cancer cell line Colo-357 we tested two more pancreatic cancer cell lines (the mouse pancreatic cancer cell line 6606 PDA and the human pancreatic cancer cell line PaTu8988T) *in vitro*. In fact similar effects could be demonstrated in both cell lines confirming the results of Colo-357. Comparable to our results in the three different cell lines Kim et al. found cell specific differences in the plasma effects, too. The colon cancer cell lines SW480 and LoVo displayed a higher resistance to plasma treatment than HCT-116 cell [19].

This effect was best seen after 10 seconds of TTP application levelling off after about 72 hours following the application. Similar effects of plasmas have been described for lung cancer cells and melanoma cells concerning apoptosis [15,20]. However, non-apoptotic cell death was not found by Kim et al. [20] applying up to 10 seconds of plasma *in vitro* whereas Keidar et al. [21] described *in vitro* findings comparable to ours. In addition, Keidar et al. did not describe any relevant damages of non-malignant cells in an *in vivo* tumour model applying 5 minutes of plasma. Yet, the exact induction mechanisms of apoptotic or non-apoptotic cell death were not analyzed. Yan et al. could recently demonstrate that the increased concentrations of nitric oxide (NO), reactive oxygen species (ROS), and lipid peroxide during the plasma treatment correlated with the increasing number of dead tumour cells [22]. Ahn et al. could demonstrate that Plasma induces apoptotic apoptosis via ROS and dysfunction of mitochondria membranes in the human cervical carcinoma cell line HeLa [23]. In contrast, cotreatment with scavengers of ROS mitigated the apoptotic effect. Also, the treatment with caspase 3 and 9 inhibitors had extenuating effects on plasma-induced cell death further confirming the involvement of mitochondria in plasma-induced apoptosis. Furthermore, a caspase 8 inhibitor had little effect on plasma-induced apoptosis, suggesting that plasma-induced apoptosis is not mediated through activation of the extrinsic apoptotic pathway involving death receptors. However, the specific step in the intrinsic apoptotic pathway affected by Plasma treatment via ROS has not been identified. Depolarization of the mitochondria membrane potential may play a central role, but further studies are necessary in this respect.

Using the TUM-CAM model we have not yet analyzed non-malignant cells but could show that solid tumour cells underwent apoptotic cell death up to a DETiP of 60 μm. These results point to a fundamental difference of the effects of TTP *in vitro* and *in vivo*. 10 seconds of TTP initially represent a sub-lethal dose of plasma *in vitro* yet initiate apoptosis in most or all tumour cells. 20 seconds of TTP induce apoptosis only in a small percentage of tumour cells irreversibly damaging the majority of tumour cells by non-apoptotic cell death. In contrast, applying TTP *in vivo* in the TUM-CAM model we have only been able to demonstrate apoptotic cell death in tumour cells so far.

Taken together our results suggest a possible application of TTP in pancreatic cancer surgery. Since we have demonstrated a DETiP of up to 60 μm leaving lower tumour layers intact not changing their rate of proliferation the treatment of solid tumours, as already suggested by other authors [21,24], may not significantly improve survival rates. Therefore, we suggest the intra-operative use of TTP in surgical patients to treat possible minimal residual disease after resection. We suggest that pancreatic cancer patients could greatly benefit from this treatment option since tumours of many patients are resectable but show minimal residual disease of retroperitoneal tissues and major supplying mesenteric vessels [3] which is an independent prognostic factor for poor survival [25]. TTP treatment could destroy these tumour cells leaving the non-malignant surrounding tissues intact since several groups have described more pronounced plasma effects on cancer cells than on stromal cell, including fibroblasts [15,20]. Keidar et al. detected “plasma selectivity” for murine melanoma cancer cells while murine macrophages were not affected [21]. However, the reason for stromal cells being more resistant to plasma treatment than cancer cells is still unknown. It remains an important part of the ongoing research of many investigators.

Furthermore, additional applications could be carried out for malignant tumours of the liver involving central non-resectable liver vessels and bile ducts as well as central lung tumours involving non-resectable vessels and/or bronchi. Finally, TTP and chemotherapeutic drugs may have synergistic effects either using low dose chemotherapies before surgery and intra-operative TTP treatment or *vice versa*[26].

## Conclusions

These promising results require the adaptation of our plasma source to an improved clinical application since the plasma plume of the kinPen09 only allows the treatment of small tumours and/or small tissue areas. A fan-like applicator would be ideal for these purposes. Also, the composition of the effective components of the TTP ought to be evaluated for their effectiveness to optimize their synergistic apoptotic actions. This applies to the modifiable components (electrons/ions, free radicals, electromagnetic particles, UV radiation and the carrier gas) of the TTP. Ongoing *in vivo* experiments will further evaluate treatment options of TTP in an orthotopic syngeneic mouse model of pancreatic cancer [17,27].

## Competing interests

The authors declare that they have no competing interests.

## Authors’ contributions

LIP: study conception and design, statistics, drafting and revision of manuscript, has read and approved the final manuscript. KE: performed histology, interpretation of data, revision of manuscript, reading and approval of final manuscript. JH: study conception and design, performed all experiments, statistics, revision of manuscript, has read and approved the final manuscript. FD: design of TUM-CAM experiments, revision of manuscript, has read and approved the final manuscript. LN: designed *in vitro* experiments, revision of manuscript, reading and approval of final manuscript. SD: interpretation of data, revision of manuscript, reading and approval of final manuscript. FUW: interpretation of data, revision of manuscript, reading and approval of final manuscript. ME: interpretation of data, revision of manuscript, reading and approval of final manuscript. NH: interpretation of data, revision of manuscript, reading and approval of final manuscript. CG: TUM-CAM experiments, revision of manuscript, has read and approved the final manuscript. CDH: interpretation of data, revision of manuscript, reading and approval of final manuscript. AK: interpretation of data, revision of manuscript, reading and approval of final manuscript. RB: design of plasma source, interpretation of data, revision of manuscript, reading and approval of final manuscript. KDW: design of plasma source, interpretation of data, revision of manuscript, reading and approval of final manuscript. OP: performed *in vitro* experiments, statistics, revision of manuscript, has read and approved the final manuscript. CB: study conception and design, revision of manuscript, has read and approved the final manuscript. WvB: study conception and design, statistics, drafting and revision of manuscript, has read and approved the final manuscript.

## Pre-publication history

The pre-publication history for this paper can be accessed here:

http://www.biomedcentral.com/1471-2407/12/473/prepub

## Supplementary Material

Additional file 1 **Table S1.** The murine cell line 6606PDA and the human cell line PaTu8988T were treated with TTP using the same set up as for Colo-357 (Table 1). 6606PDA as well as PaTu8988T showed a significant increase in cell death after TTP treatment (p=0.0303 and p=0.0043, respectively). Click here for file

Additional file 2 **Figure S1.** Cell cycle analyses: Percentage of apoptosis as sub-diploid peaks left to G0/G1-peak. Apoptotic cells 72 hours after TTP *versus* control and argon gas; example of 5 independent experiments. Click here for file
