# Supplementary figures and images for: Tissue Tolerable Plasma (TTP) induces apoptosis in pancreatic cancer cells in vitro and in vivo
Source: BMC Cancer. 2012 Oct 15;12:473. doi: 10.1186/1471-2407-12-473 (PMC3598726; doi:10.1186/1471-2407-12-473)

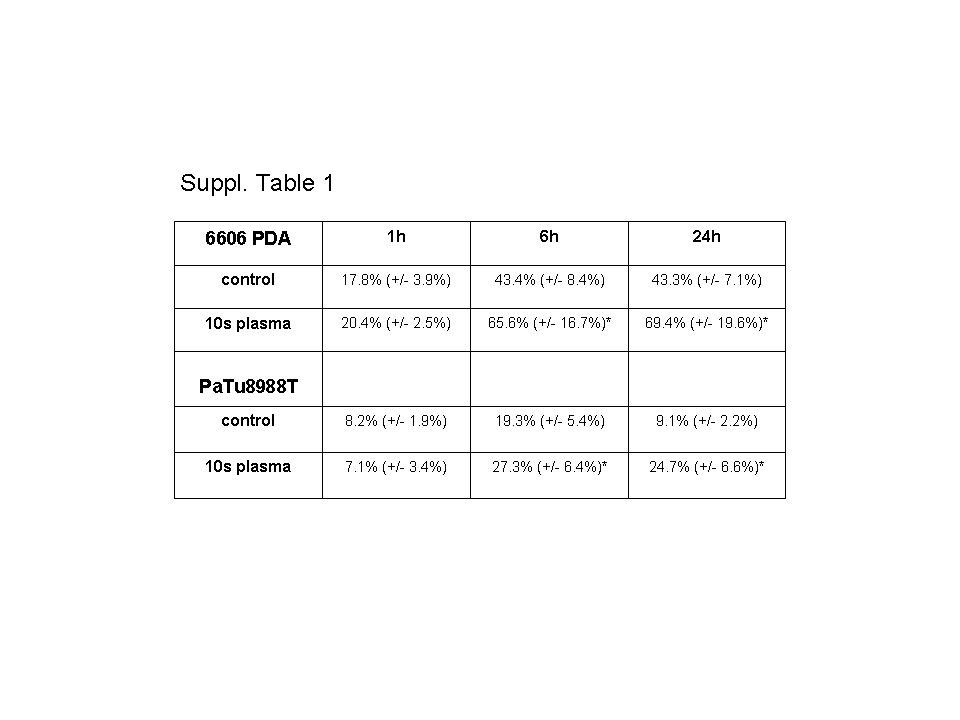

Supplement: Additional file 1 — Table S1. The murine cell line 6606PDA and the human cell line PaTu8988T were treated with TTP using the same set up as for Colo-357 (Table 1). 6606PDA as well as PaTu8988T showed a significant increase in cell death after TTP treatment (p=0.0303 and p=0.0043, respectively). [file 1471-2407-12-473-S1.tiff]

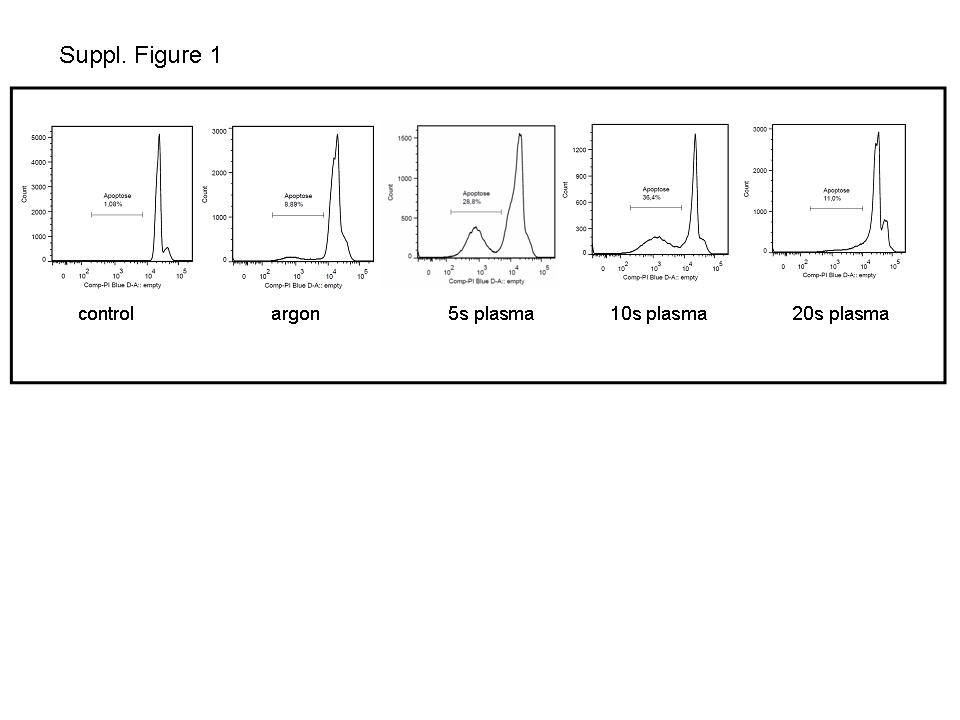

Supplement: Additional file 2 — Figure S1. Cell cycle analyses: Percentage of apoptosis as sub-diploid peaks left to G0/G1-peak. Apoptotic cells 72 hours after TTP versus control and argon gas; example of 5 independent experiments. [file 1471-2407-12-473-S2.jpeg]
